# Evaluation of the Clearview^®^ Malaria pLDH as malaria rapid diagnostic test in travellers

**DOI:** 10.1186/1475-2875-9-S2-P15

**Published:** 2010-10-20

**Authors:** Sandrine Houzé, Véronique Hubert, Pressler D Cohen, Baruch Rivetz, Jacques Le Bras

**Affiliations:** 1 Laboratory of Parasitology-French Malaria Reference Center, AP-HP Bichat Hospital, Paris, 75018, France; 2 Orgenics Ltd, Yavne, 70650, Israel

## Background

Malaria Rapid Diagnostic Tests (RDTs) are widely used to diagnose malaria. The present prospective study evaluated a new RDT, the Clearview^®^ Malaria pLDH test targeting the pan*-Plasmodium* antigen lactate dehydrogenase (pLDH).

## Methods

The Clearview^®^ Malaria pLDH test was evaluated on fresh samples obtained in returned international travellers using microscopy corrected by PCR as the reference method. Included samples were *P. falciparum* (139), *P. vivax* (22), *P. ovale* (20), *P. malariae* (7), and 102 negative.

## Results

Overall sensitivity for the detection of *Plasmodium sp.* was 93.2%. For *P. falciparum,* the sensitivity was 98.6%; for *P. vivax, P. ovale* and *P. malariae,* overall sensitivities were 90.9%, 60.0% and 85.7% respectively. For *P. falciparum* and for *P. vivax,* the sensitivities increased to 100% at parasite densities above 100/μl. The specificity was 100%. The test was easy to perform and the result was stable for at least 1 hour.

## Conclusion

The Clearview^®^ Malaria pLDH was efficient for the diagnosis of malaria. The test was very sensitive for *P. falciparum* and *P. vivax* detection. The sensitivities for *P. ovale* and *P. malariae* were better than other RDTs [1].
